# Multiple laryngeal neuromas: rare benign tumor

**DOI:** 10.31744/einstein_journal/2019AI4465

**Published:** 2019-02-28

**Authors:** Altair da Silva Costa, Iunis Suzuki, Addy Lidvina Mejia Palomino, Marcelo Gervilla Gregorio, Paulo Rogerio Scordamaglio, Marcia Jacomelli

**Affiliations:** 1 Hospital Israelita Albert Einstein, São Paulo, SP, Brazil.; 2 Hospital das Clínicas, Faculdade de Medicina, Universidade de São Paulo, São Paulo, SP, Brazil.

**Figure 1:**
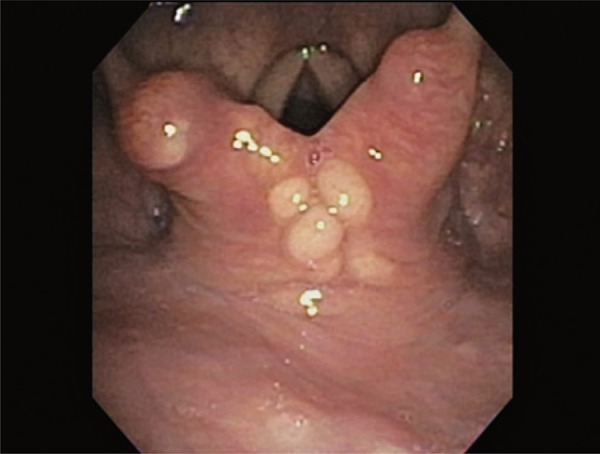
Lesions in the posterior region of larynx

**Figure 2:**
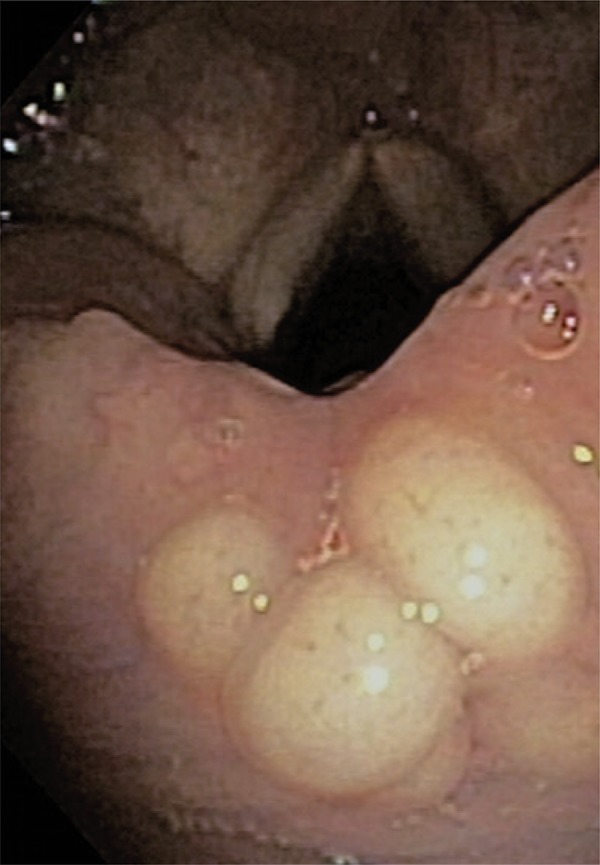
Detailed image of lesions in the posterior region of larynx

**Figure 3:**
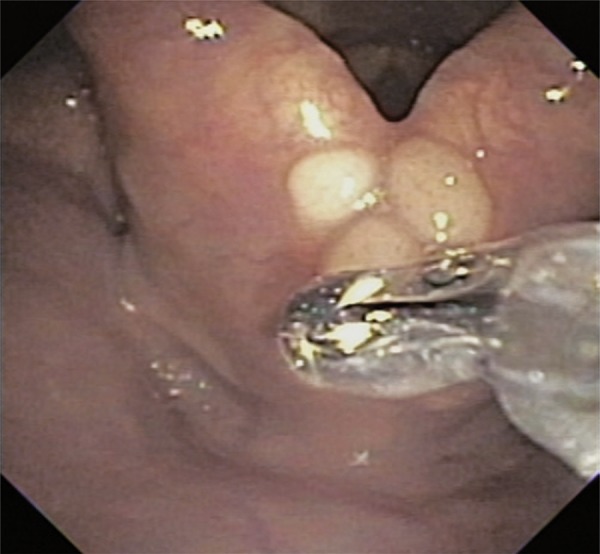
Biopsy of lesions in the posterior region

A 66-year old female patient complained of sensation of a foreign body in her pharynx and of dry cough for the past 6 months. She denied dysphonia, dysphagia or dyspnea. Endoscopy was performed for suspected gastroesophageal reflux, and it showed several roundish, yellowish and well-limited lesions, in the posterior region of the larynx, adjacent to the arytenoid cartilages ( Figures 1 and 2 ). A biopsy of the lesions was performed ( Figure 3 ), and the pathological examination revealed neoplasm with clear limits, composed of a diffuse neural proliferation, with thin, tortuous and disorganized fibers and no atypia. No mitoses, cysts or necrosis were observed.

The immunohistochemistry examination showed diffuse positivity for protein S100 (present in neural crest-derived cells) and CD34 (marker of hematopoietic and lymphoid tissues and vascular endothelium). The epithelial membrane antigen (EMA) was negative, and compatible with multiple mucosal neuromas in the larynx.

The exam is available in video recording at https://youtu.be/uUs5p5qWjQg.

Among the benign laryngeal lesions, neural neoplasms are rare. This group includes the peripheral nerve sheath tumors, which are basically formed by some of these three cell types: Schwann cells, fibroblasts and perineural cells, with or without axons.^(^ ^1^ ^,^ ^2^ ^)^ Schwann cells are mainly related to three types of tumors: neuromas, schwannomas and neurofibromas, which are differentiated by their morphological pattern and proportions of their diverse cell components.^(^ ^1^ ^-^ ^3^ ^)^

The solitary circumscribed neuroma, or palisaded encapsulated neuroma, is defined as a neoformation originating from a nerve fiber, with variable amounts of all usual components of a peripheral nerve. It was identified as a disease by Reed, in 1972, but later, in 1994, Megahed described its immunohistochemistry characteristics.^(^ ^1^ ^)^ The differential diagnoses include traumatic neuroma, neurofibroma, neurilemoma (or schwannoma) and leiomyoma. Protein S-100 is an immunohistochemistry marker widely used to identify neoplasms of neural origin.^(^ ^1^ ^-^ ^4^ ^)^

Laryngeal multiple mucosal neuromas are rare and can be associated with the clinical syndrome “multiple endocrine neoplasia type 2B” (MEN 2B).^(^ ^5^ ^-^ ^8^ ^)^ Multiple endocrine neoplasia can be type 1 (involving the parathyroid glands, pancreas and hypophysis) or 2A and 2B. The genetic syndrome MEN 2A is characterized by presence of medullary carcinoma of thyroid (95%), pheochromocytoma (30 to 50%) and hyperparathyroidism (10 to 20%). Multiple endocrine neoplasia 2B is characterized by medullary carcinoma of thyroid (90%), pheochromocytoma (45%), ganglioneuromatosis (100%) and marfanoid habitus (65%).^(^ ^6^ ^)^

Neuromas are rare, and occur between the third and fifth decades of life, with no distinction between gender.^(^ ^1^ ^,^ ^2^ ^)^ Ninety percent of them are on the facial skin, and the mucosal location is not common; the laryngeal neuromas are even rarer. Like other neural lesions, its relation with clinically relevant syndromes has been investigated, such as neurofibromatosis or multiple endocrine neoplasia, but a clear association has not been identified. The treatment is surgical resection.^(^ ^1^ ^,^ ^4^ ^,^ ^7^ ^,^ ^8^ ^)^
